# Supplementary figures and images for: Maturation of HIV-1 neutralizing antibodies in a germinal center conditional expression mouse model
Source: PLoS Pathog. 2026 Jun 22;22(6):e1014373. doi: 10.1371/journal.ppat.1014373 (PMC13313368; doi:10.1371/journal.ppat.1014373)

**S1 Fig**

**A**

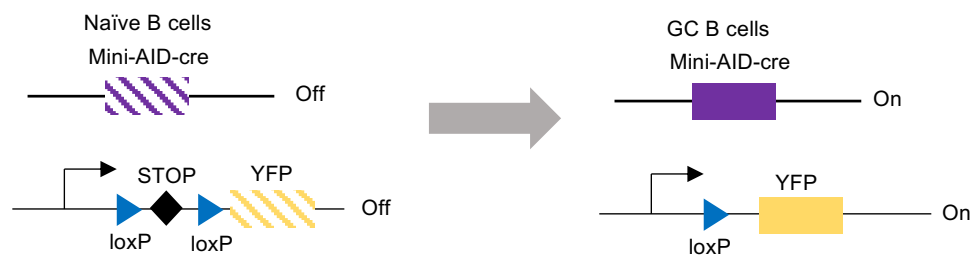

**B**

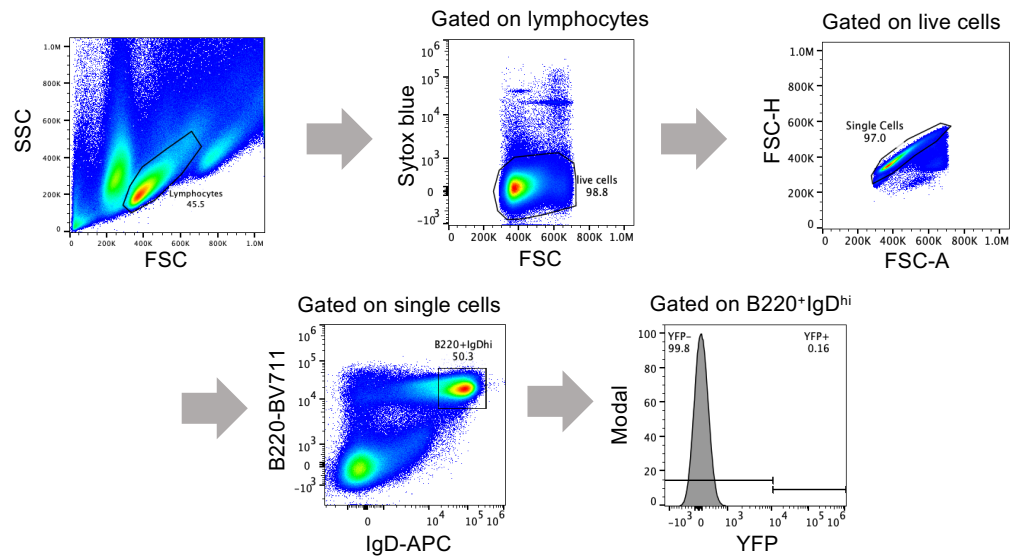

**C**

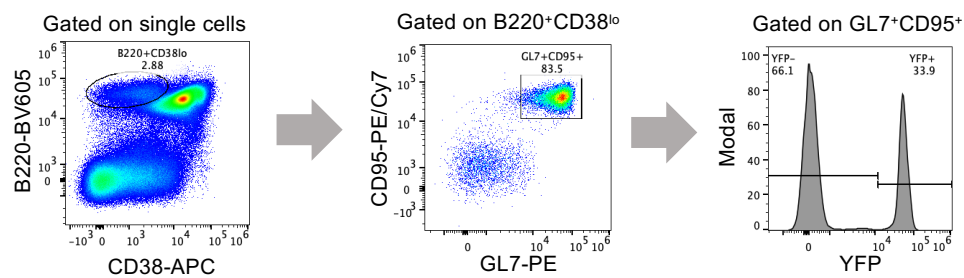

Supplement: S1 Fig — (A) Diagram of the YFP reporter assay. (B) Flow cytometric analysis of YFP expression in naive B cells of unimmunized mouse. Gating scheme: lymphocyte>live cell>single cell>B220+IgDhi (naive B cell)>YFP histogram. Events displayed: FSC/SSC (2.00x10^6^), FSC/Sytox blue (9.09x10^5^), FSC-A/FSC-H (8.99x10^5^), IgD/B220 (8.72x10^5^), YFP histogram (4.38x10^5^). (C) Flow cytometric analysis of YFP expression in GC B cells after immunization with sheep red blood cells. Gating scheme: lymphocyte>live cell>single cell>B220+CD38lo>GL7+CD95+>YFP histogram. Events displayed: CD38/B220 (6.33x10^5^), GL7/CD95 (18221), YFP histogram (15213). (PDF) [file ppat.1014373.s001.pdf]

**S5 Fig**

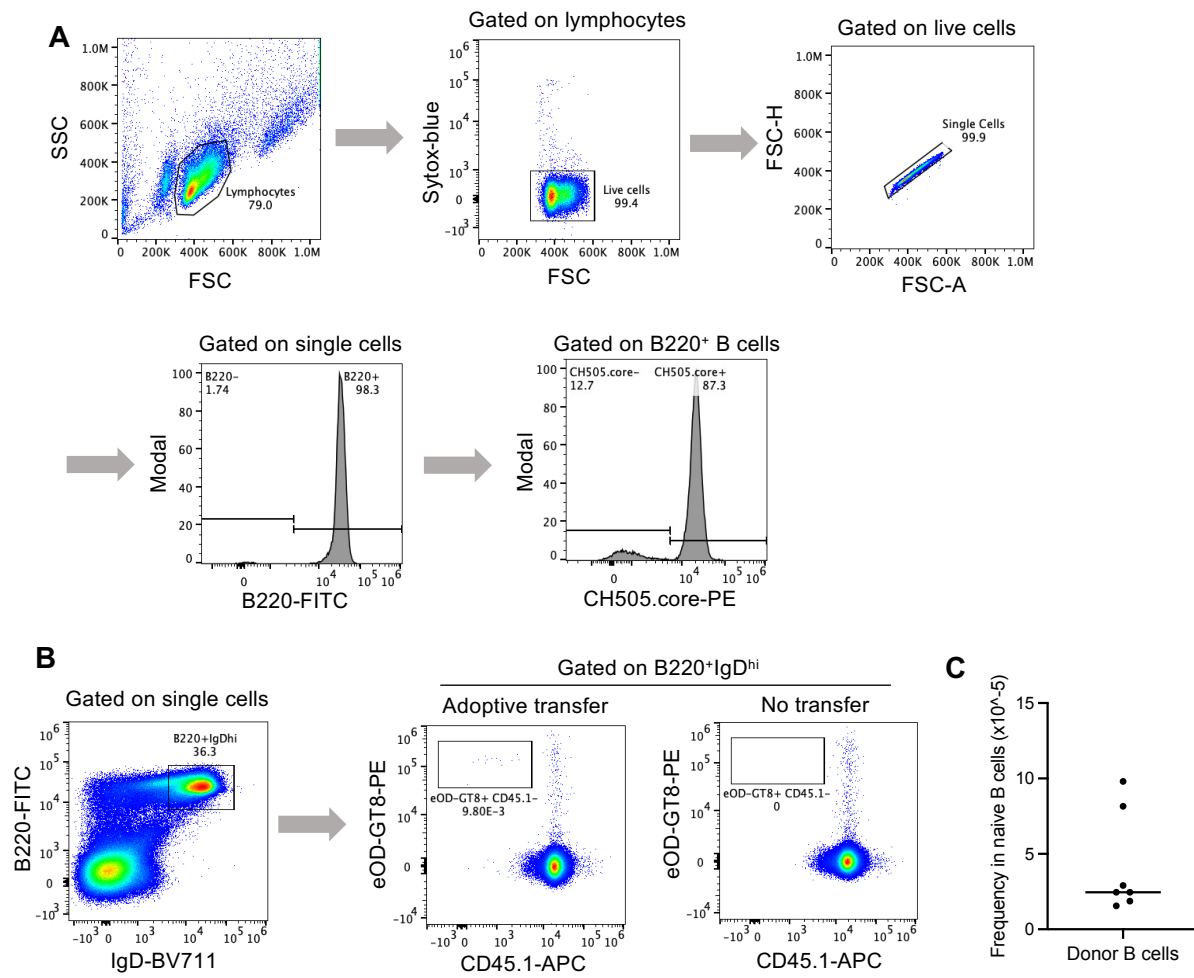

Supplement: S5 Fig — (A) Flow cytometric analysis of donor splenic B cells from the GC model. Splenic B cells from the GC model were purified with MACS. The FACS plots show the analysis of the purified splenic B cells before adoptive transfer. Gating scheme: lymphocyte>live cell>single cell>B220+ B cell>CH505.core+ VRC01 B cell. Events displayed: FSC/SSC (49041), FSC/Sytox blue (38719), FSC-A/FSC-H (38492), B220 histogram (38453), CH505.core histogram (37784). (B) Flow cytometric analysis of donor B cells in the naive B cell compartment of adoptively transferred mice. The analysis was done on splenocytes two days after adoptive transfer. Gating scheme: lymphocyte>live cell>single cell>B220+IgDhi (naive B cell)>CD45.1-eOD-GT8+ (donor B cell). Events displayed: IgD/B220 (5.26x10^5^), CD45.1/eOD-GT8 Adoptive transfer (142831), No transfer (190796). (C) Summary of donor B cell frequency in naive B cell compartment in adoptively transferred mouse. The dot plot is based on FACS analysis as shown in panel B. Each dot corresponds to one adoptively transferred mouse; the line represents the median. (PDF) [file ppat.1014373.s005.pdf]

**S6 Fig**

**A**

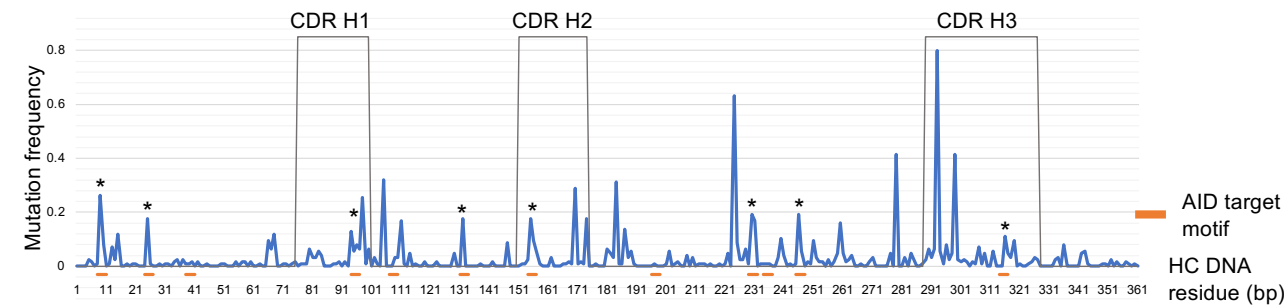

**B**

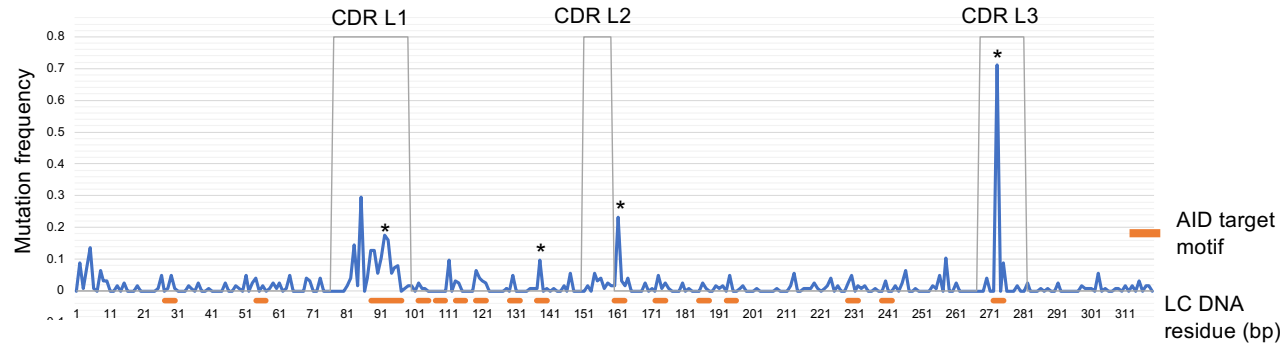

Supplement: S6 Fig — The blue curve shows mutation frequencies at each DNA residue of the IA-VRC01 HC (A) and LC (B) after immunization. The residue number is indicated at the bottom. The orange bars represent AID target motif (RGYW or WRCY). Mutation peaks (mutation frequency > 0.1) that coincide with AID target motif are marked with *. (PDF) [file ppat.1014373.s006.pdf]

**S7 Fig**

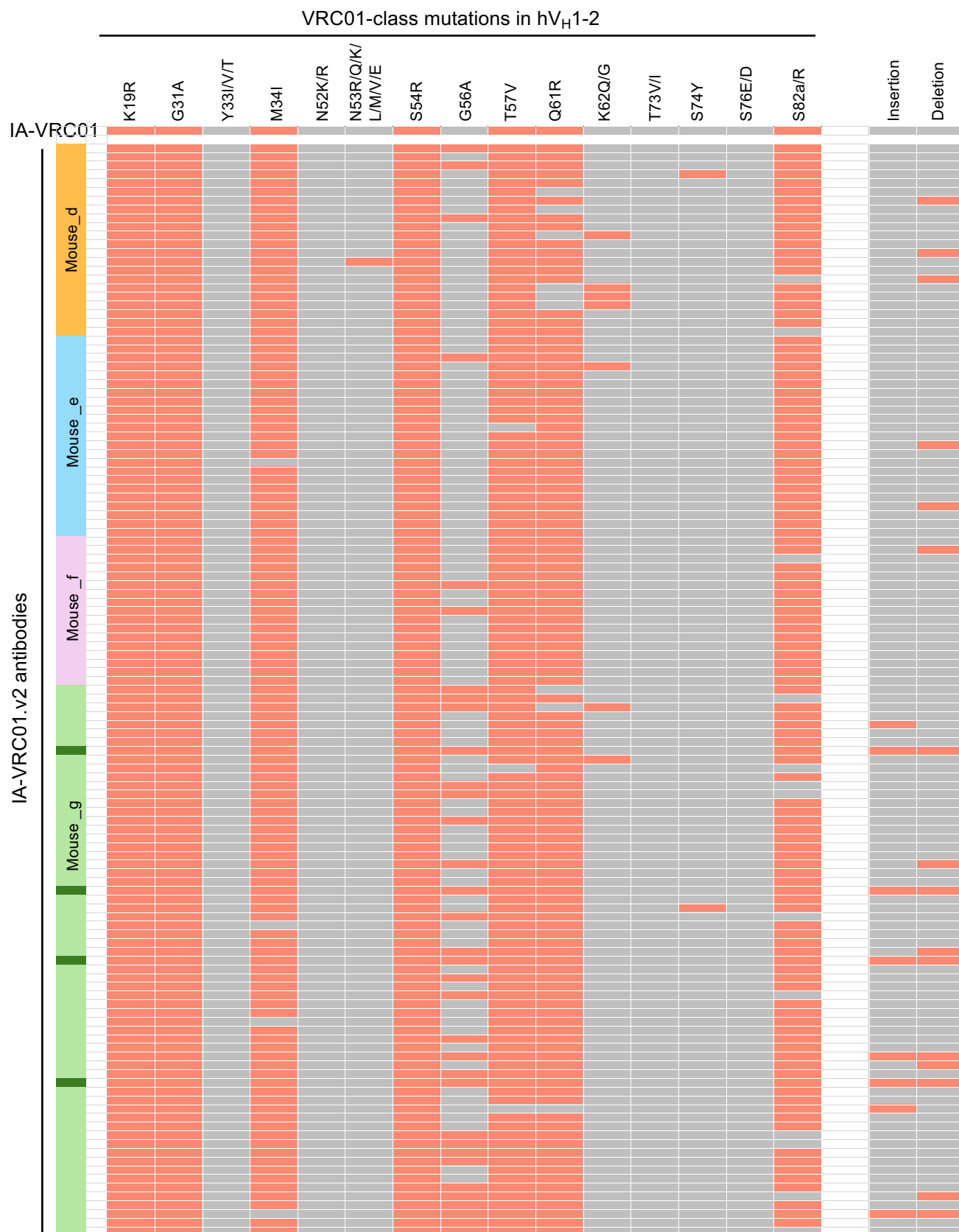

Supplement: S7 Fig — VRC01 class mutations in the hVH1-2 segment and indels are listed on top; the presence or absence of VRC01 class mutations and indels are represented by red and grey respectively. Each horizontal line represents one antibody. IA-VRC01 is represented by the line at the top. Below are antibodies after boost immunization (IA-VRC01.v2), isolated from 4 mice, and the mouse origin of each antibody is indicated to the left. The mutation status in Fig 6B is based on this plot. (PDF) [file ppat.1014373.s007.pdf]
