# Supplementary material for: Maturation of HIV-1 neutralizing antibodies in a germinal center conditional expression mouse model
Source: PLoS Pathog. 2026 Jun 22;22(6):e1014373. doi: 10.1371/journal.ppat.1014373 (PMC13313368; doi:10.1371/journal.ppat.1014373)
Supplement: S2 Fig — A) VRC01HC alignment. The first and second rows are the DNA sequences of the GL-VRC01 and IA-VRC01. Differences between GL-VRC01 and IA-VRC01 are marked with * on top. The sequence of Naive_VRC01HC is the logo plot of all the Sanger sequencing results of sc-RT-PCR products from naive B cells of the GC model (Fig 3C). The height of the residue at each position correlates with its frequency. Prime_VRC01HC is the logo plot of VRC01HC sequences from sorted donor GC B cells after prime immunization of adoptively transferred mice (Fig 4B and 4D). Boost_VRC01HC is the logo plot of VRC01HC sequences from sorted GC B cells after two boost immunizations (Fig 4G). The heterogeneity of residues at some positions reflects somatic hypermutation. (B) VRC01LC alignment. (PDF) [file ppat.1014373.s002.pdf]

S2 Fig

A

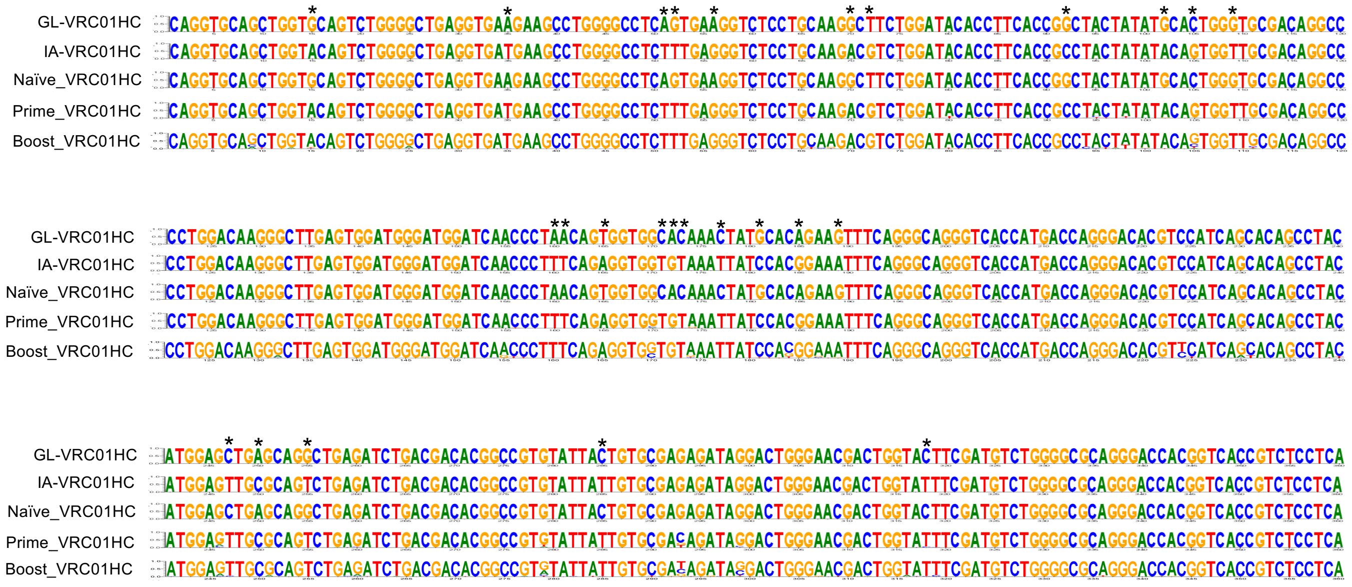
GL-VRC01HC
IA-VRC01HC
Naïve_VRC01HC
Prime_VRC01HC
Boost_VRC01HC

B

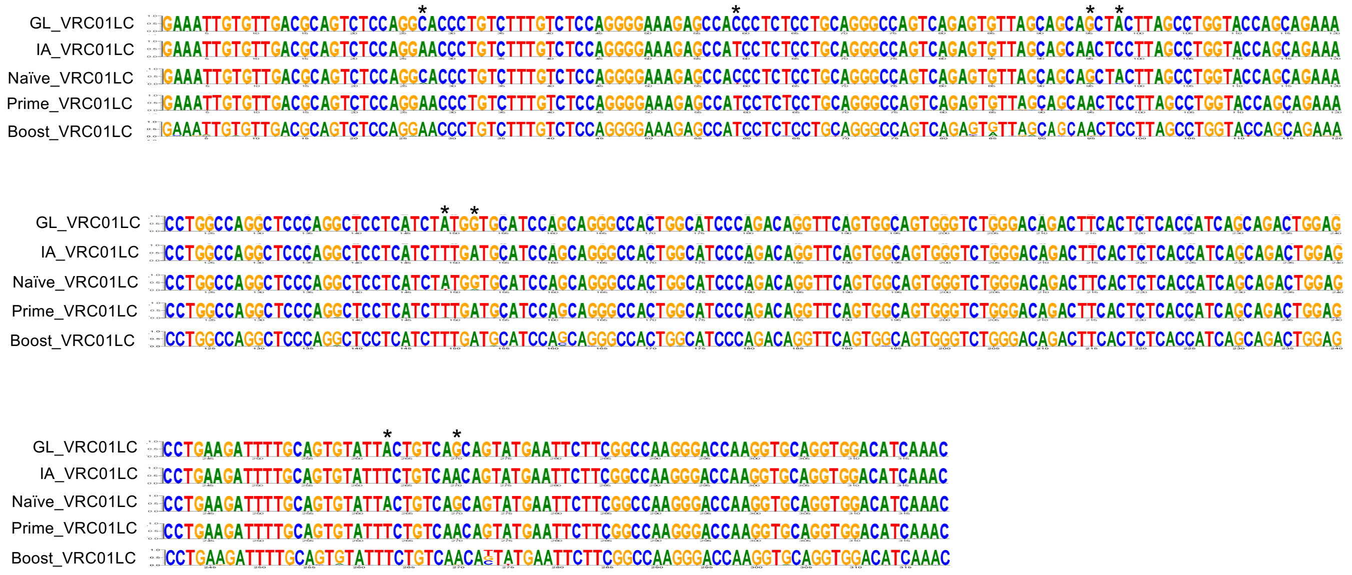
GL_VRC01LC
IA_VRC01LC
Naïve_VRC01LC
Prime_VRC01LC
Boost_VRC01LC
