# Supplementary material for: Maturation of HIV-1 neutralizing antibodies in a germinal center conditional expression mouse model
Source: PLoS Pathog. 2026 Jun 22;22(6):e1014373. doi: 10.1371/journal.ppat.1014373 (PMC13313368; doi:10.1371/journal.ppat.1014373)
Supplement: S3 Fig — (A) Analysis of VRC01HC from sorted naive B cells of unimmunized GC model (Fig 3C). The diagram shows the position of the PCR primers (arrows) for amplifying VRC01HC cDNA. Due to somatic hypermutation, IA-VRC01HC differs from GL-VRC01HC in RsaI restriction digest pattern. The gel image below is an example of sc-RT-PCR products that have been digested with RsaI. Each lane contains the PCR product of a single cell. Sc-RT-PCR did not work for the cells in empty lanes. (B) Analysis of VRC01LC from sorted naive B cells of unimmunized GC model (Fig 3C). The panel is analogous to panel A. IA-VRC01LC differs from GL-VRC01LC in NsiI restriction digest pattern. (C) Analysis of VRC01HC from donor GC B cells after prime immunization (Fig 4B and 4D). (D) Analysis of VRC01LC from donor GC B cells after prime immunization (Fig 4B and 4D). (PDF) [file ppat.1014373.s003.pdf]

S3 Fig

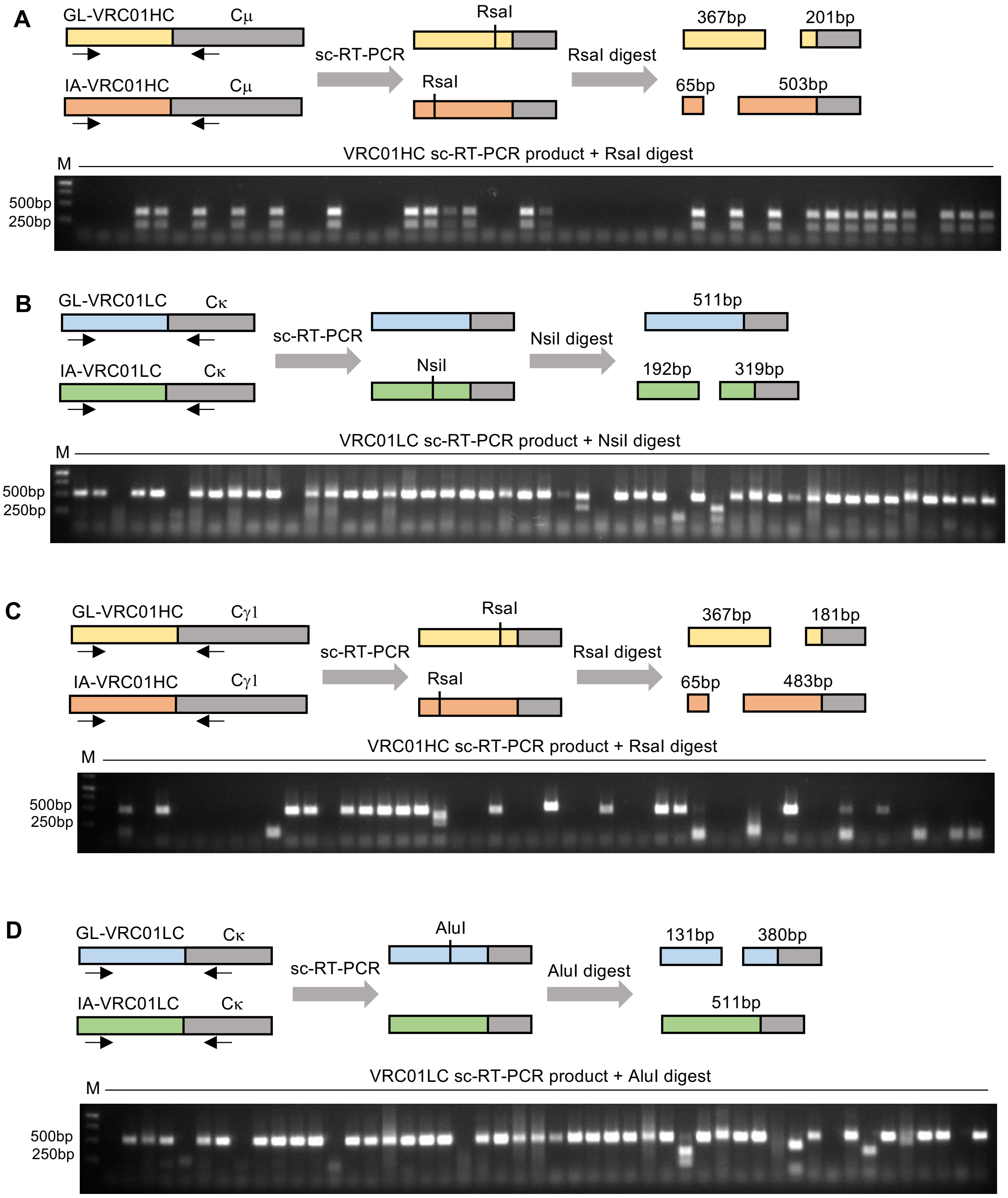

A
GL-VRC01HC
Cμ
IA-VRC01HC
Cμ
sc-RT-PCR
RsaI
RsaI
RsaI digest
367bp
201bp
65bp
503bp
M
VRC01HC sc-RT-PCR product + RsaI digest
500bp
250bp
B
GL-VRC01LC
Cκ
IA-VRC01LC
Cκ
sc-RT-PCR
NsiI
NsiI digest
511bp
192bp
319bp
M
VRC01LC sc-RT-PCR product + NsiI digest
500bp
250bp
C
GL-VRC01HC
Cγ1
IA-VRC01HC
Cγ1
sc-RT-PCR
RsaI
RsaI
RsaI digest
367bp
181bp
65bp
483bp
M
VRC01HC sc-RT-PCR product + RsaI digest
500bp
250bp
D
GL-VRC01LC
Cκ
IA-VRC01LC
Cκ
sc-RT-PCR
AluI
AluI digest
131bp
380bp
511bp
M
VRC01LC sc-RT-PCR product + AluI digest
500bp
250bp
