# Supplementary material for: Maturation of HIV-1 neutralizing antibodies in a germinal center conditional expression mouse model
Source: PLoS Pathog. 2026 Jun 22;22(6):e1014373. doi: 10.1371/journal.ppat.1014373 (PMC13313368; doi:10.1371/journal.ppat.1014373)

S4 Fig

A

		Sanger sequencing					Restriction digest			
		Mouse #1	Mouse #2	Mouse #3	Mouse #4	Mouse #5	Mouse #1	Mouse #6	Total HC/LC	Frequency
HC	GL-VRC01HC	23	49	24	24	24	41	53	238	1.00
	IA-VRC01HC	0	0	0	0	0	0	0	0	0.00
LC	GL-VRC01LC	23	39	24	24	24	41	53	228	0.96
	IA-VRC01LC	0	10	0	0	0	0	0	10	0.04

B

		Sanger sequencing			Restriction digest			
		Mouse #1	Mouse #2	Mouse #3	Mouse #1	Mouse #2	Total HC/LC	Frequency
HC	GL-VRC01HC	0	0	0	0	0	0	0.000
	IA_VRC01HC	17	18	18	29	26	108	1.000
LC	GL-VRC01LC	0	1	0	0	0	1	0.009
	IA-VRC01LC	17	17	18	29	26	107	0.991

C

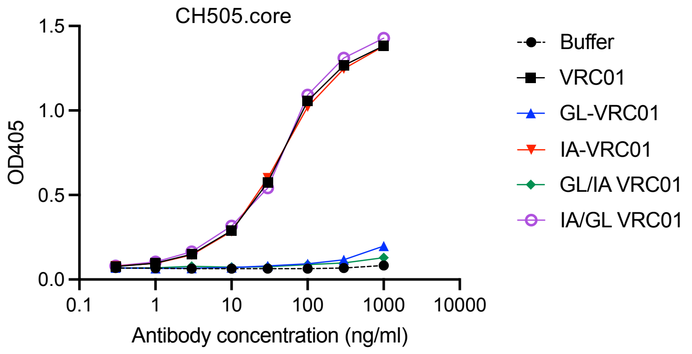

Supplement: S4 Fig — (A) sc-RT-PCR results of naive B cells. This table is based on the experiments in Figs 3C, S2 and S3. The table includes more details than the one in Fig 3C. (B) sc-RT-PCR results of donor GC B cells after prime immunization. This table is based on the experiments in Figs 4B, 4D, S2 and S3. This table includes more details than the one in Fig 4D. (C) Analysis of the binding activities of hybrid antibodies (GL/IA-VRC01, IA/GL-VRC01) by ELISA. (PDF) [file ppat.1014373.s004.pdf]
