# Supplementary material for: Maturation of HIV-1 neutralizing antibodies in a germinal center conditional expression mouse model
Source: PLoS Pathog. 2026 Jun 22;22(6):e1014373. doi: 10.1371/journal.ppat.1014373 (PMC13313368; doi:10.1371/journal.ppat.1014373)
Supplement: S8 Fig — Antibody 8g was the top Env binder among the elicited antibodies (IA-VRC01.v2, Fig 6). In antibody 8g.1, the 2aa insertion in CDR H3 was removed. In antibody 8g.3, the 2aa CDR L1 deletion was restored to the original sequence. Binding activity was quantified by the AUC of ELISA titration curve. The table shows the ratio of Env binding activities of the two revertants relative to the original antibody 8g; the binding activity of 8g was set as 1. (PDF) [file ppat.1014373.s008.pdf]

S8 Fig

	8g	8g.1	8g.3
426.c	1.00	0.75	0.68
CH505	1.00	0.62	0.93
CH848	1.00	0.48	0.91
QJX41594.1	1.00	0.05	0.93
JRFL	1.00	0.75	0.91
AZ172138.1	1.00	0.90	0.92
YU2	1.00	0.28	0.87
ZM106.9	1.00	0.44	0.59

>0.75
0.5-0.75
0.25-0.5
0.05-0.25
<0.05
