# Supplementary material for: Maturation of HIV-1 neutralizing antibodies in a germinal center conditional expression mouse model
Source: PLoS Pathog. 2026 Jun 22;22(6):e1014373. doi: 10.1371/journal.ppat.1014373 (PMC13313368; doi:10.1371/journal.ppat.1014373)

S9 Fig

A

		IA-VRC01		8g		26g		41g		VRC01	
**Virus ID**	**Clade**	**IC50**	**MPI**	**IC50**	**MPI**	**IC50**	**MPI**	**IC50**	**MPI**	**IC50**	**MPI**
6535.3	B	>50	24	>50	22	>50	32	>50	19	3.028	99
QH0692.42	B	>50	20	>50	30	>50	14	>50	19	1.37	99
SC422661.8	B	>50	7	>50	9	>50	14	>50	8	0.15	100
PVO.4	B	>50	0	5.512	91	>50	45	9.156	83	0.819	100
TRO.11	B	>50	7	3.379	98	>50	0	9.77	86	0.821	100
AC10.0.29	B	>50	0	>50	0	>50	0	>50	0	1.732	99
RHPA4259.7	B	>50	1	25.674	59	>50	42	49.407	50	0.032	100
THRO4156.18	B	>50	21	33.588	58	>50	9	>50	32	8.949	89
REJO4541.67	B	>50	0	>50	0	>50	0	>50	0	0.126	100
TRJO4551.58	B	>50	0	>50	0	>50	7	>50	0	0.184	100
WITO4160.33	B	>50	0	>50	13	>50	17	>50	11	0.07	100
CAAN5342.A2	B	>50	14	>50	4	>50	16	>50	13	1.401	100
WEAU_d15_410_787	B (T/F)	>50	22	>50	15	>50	29	>50	15	0.08	100
1006_11_C3_1601	B (T/F)	>50	20	3.337	86	>50	48	7.744	79	0.157	99
1054_07_TC4_1499	B (T/F)	>50	14	>50	13	>50	17	>50	22	1.209	99
1056_10_TA11_1826	B (T/F)	>50	12	>50	16	>50	19	>50	8	0.774	100
1012_11_TC21_3257	B (T/F)	>50	8	>50	0	>50	20	>50	2	0.166	100
6240_08_TA5_4622	B (T/F)	>50	26	22.793	70	>50	32	44.615	53	0.692	100
6244_13_B5_4576	B (T/F)	>50	11	17.561	74	>50	19	>50	28	0.459	100
62357_14_D3_4589	B (T/F)	1.133	98	0.335	100	0.878	100	0.383	100	2.456	92
SC05_8C11_2344	B (T/F)	>50	23	>50	18	>50	18	>50	16	0.805	100
Du156.12	C	>50	27	>50	33	>50	44	>50	28	0.13	100
Du172.17	C	>50	26	>50	21	>50	14	>50	12	>50	18
Du422.1	C	>50	16	>50	21	>50	11	>50	24	>50	27
ZM197M.PB7	C	>50	12	>50	10	>50	13	>50	15	0.72	100
ZM214M.PL15	C	>50	0	1.737	85	>50	34	8.075	79	1.395	100
ZM233M.PB6	C	>50	15	>50	9	>50	12	>50	5	2.747	97
ZM249M.PL1	C	>50	15	>50	12	>50	32	>50	11	0.093	100
ZM53M.PB12	C	>50	0	9.327	84	>50	41	9.639	82	1.22	100
ZM109F.PB4	C	>50	7	>50	15	>50	11	>50	0	0.219	100
ZM135M.PL10a	C	>50	7	>50	13	>50	9	>50	14	1.771	97
CAP45.2.00.G3	C	>50	0	>50	0	>50	0	>50	0	12.664	78
CAP210.2.00.E8	C	>50	17	>50	10	>50	9	>50	14	>50	18
HIV-001428-2.42	C	>50	11	>50	7	48.734	53	>50	0	0.016	100
HIV-0013095-2.11	C	>50	21	>50	16	>50	0	>50	31	0.235	100
HIV-16055-2.3	C	>50	7	3.279	91	28.147	65	2.469	95	0.158	100
HIV-16845-2.22	C	>50	0	11.798	73	>50	17	23.46	62	4.949	90
Ce1086_B2	C (T/F)	>50	24	0.803	97	33.548	60	3.234	93	0.568	100
Ce0393_C3	C (T/F)	>50	0	>50	3	>50	0	>50	0	1.544	100
Ce1176_A3	C (T/F)	>50	0	>50	39	>50	6	>50	30	2.242	98
Ce2010_F5	C (T/F)	>50	8	>50	19	>50	12	>50	15	0.865	100
Ce0682_E4	C (T/F)	>50	0	0.209	99	5.029	76	0.58	93	0.102	100
Ce1172_H1	C (T/F)	>50	6	>50	18	>50	10	>50	11	>50	12
Ce2060_G9	C (T/F)	>50	9	7.426	84	>50	33	41.062	56	0.484	97
Ce703010054_2A2	C (T/F)	>50	22	>50	25	>50	8	>50	23	1.111	100
BF1266.431a	C (T/F)	>50	4	>50	0	>50	31	>50	0	0.048	100
246F C1G	C (T/F)	>50	2	>50	11	>50	7	>50	9	4.683	85
249M B10	C (T/F)	>50	6	>50	16	>50	0	>50	7	1.192	100
ZM247v1(Rev-)	C (T/F)	>50	0	>50	11	>50	0	>50	2	0.461	99
7030102001E5(Rev-)	C (T/F)	>50	4	>50	0	>50	0	>50	6	0.921	100
1394C9G1(Rev-)	C (T/F)	>50	12	>50	44	>50	17	>50	34	0.395	97
Ce704809221_1B3	C (T/F)	>50	12	>50	46	>50	36	>50	45	0.452	100
CNE19	BC	>50	9	2.429	86	48.345	51	5.142	79	0.153	100
CNE20	BC	>50	6	>50	10	>50	6	>50	13	14.196	62
CNE21	BC	>50	11	>50	29	>50	15	>50	9	0.272	100
CNE17	BC	>50	3	>50	6	>50	6	>50	20	0.762	100
CNE30	BC	>50	15	10.848	81	>50	23	16.091	72	0.752	100
CNE52	BC	>50	10	39.835	58	>50	10	>50	17	0.163	100
CNE53	BC	>50	47	0.361	99	1.383	94	0.421	97	0.122	100
CNE58	BC	>50	5	1.03	100	7	90	1.265	99	0.374	100
MS208.A1	A	>50	20	18.936	69	>50	35	25.294	64	0.218	100
Q23.17	A	>50	22	2.015	99	2.879	95	1.437	99	0.138	100
Q461.e2	A	>50	23	26.957	60	>50	21	>50	47	0.467	100
Q769.d22	A	>50	11	0.257	100	0.627	98	0.321	100	0.03	100
Q259.d2.17	A	>50	21	0.208	99	47.389	53	15.104	69	0.081	100
Q842.d12	A	>50	10	40.05	58	40.278	58	27.118	59	0.036	100
0260.v5.c36	A	>50	17	3.134	97	8.692	86	3.319	95	0.449	100
3415.v1.c1	A	>50	17	>50	21	>50	27	>50	13	0.097	100
3365.v2.c2	A	>50	8	2.134	95	9.821	80	2.533	94	0.119	100
191955_A11	A (T/F)	>50	23	>50	19	>50	23	>50	18	1.581	93
191084 B7-19	A (T/F)	>50	21	>50	28	>50	25	>50	18	0.167	100
9004SS_A3_4	A (T/F)	>50	30	8.432	81	>50	38	17.376	68	0.272	100
T257-31	CRF02_AG	>50	18	>50	33	>50	22	>50	22	3.195	97
928-28	CRF02_AG	>50	26	>50	25	>50	32	>50	22	0.687	100
263-8	CRF02_AG	>50	13	4.732	83	28.472	59	5.222	83	0.346	100
T250-4	CRF02_AG	>50	15	>50	18	>50	11	>50	15	>50	12
T251-18	CRF02_AG	>50	15	>50	15	>50	5	>50	10	5.035	95
T278-50	CRF02_AG	>50	26	>50	24	>50	26	>50	25	>50	23
T255-34	CRF02_AG	>50	5	>50	45	>50	16	>50	31	0.867	100
211-9	CRF02_AG	>50	14	>50	20	>50	23	>50	18	14.189	81
235-47	CRF02_AG	>50	22	>50	22	>50	45	>50	13	0.048	100
620345.c01	CRF01_AE	>50	14	>50	18	>50	13	>50	6	>50	11
CNE8	CRF01_AE	>50	12	>50	27	>50	17	>50	11	0.555	100
C1080.c03	CRF01_AE	>50	18	>50	21	>50	13	>50	3	5.437	90
R2184.c04	CRF01_AE	>50	18	>50	9	>50	22	>50	5	0.217	100
R1166.c01	CRF01_AE	>50	8	>50	44	>50	32	36.631	59	1.999	99
R3265.c06	CRF01_AE	>50	0	>50	21	>50	0	>50	9	1.516	99
C2101.c01	CRF01_AE	>50	0	1.55	95	19.789	62	1.593	96	0.344	100
C3347.c11	CRF01_AE	0.472	99	0.085	100	0.543	100	0.113	100	0.12	100
C4118.c09	CRF01_AE	>50	7	>50	22	>50	20	>50	18	0.268	100
CNE5	CRF01_AE	>50	0	>50	0	>50	0	>50	0	0.405	100
BJOX009000.02.4	CRF01_AE	>50	11	>50	8	>50	10	>50	11	2.335	98
BJOX015000.11.5	RF01_AE (T/F	>50	0	>50	27	>50	0	>50	18	0.802	100
BJOX010000.06.2	RF01_AE (T/F	>50	8	>50	5	>50	8	>50	12	10.691	84
BJOX025000.01.1	RF01_AE (T/F	>50	18	0.438	100	1.6494	96	1	99	18.392	73
BJOX028000.10.3	RF01_AE (T/F	>50	0	>50	0	>50	0	>50	0	0.405	97
X1193_c1	G	>50	0	>50	0	>50	2	>50	0	0.133	100
P0402_c2_11	G	>50	6	>50	14	>50	5	>50	1	0.204	100
X1254_c3	G	>50	9	>50	12	>50	33	>50	4	0.102	100
X2088_c9	G	>50	0	>50	0	>50	0	>50	0	>50	1
X2131_C1_B5	G	>50	8	>50	12	>50	12	>50	0	1.203	100
P1981_C5_3	G	>50	0	>50	0	>50	0	>50	0	0.42	100
X1632_S2_B10	G	>50	0	>50	0	>50	0	>50	0	0.312	89
3016.v5.c45	D	>50	14	>50	5	>50	20	>50	15	0.19	100
A07412M1.vrc12	D	>50	9	11.487	82	>50	28	>50	45	0.241	100
231965.c01	D	>50	8	>50	12	>50	8	>50	0	0.537	100
231966.c02	D	>50	1	>50	5	>50	9	>50	0	0.314	100
6405.v4.c34	D	>50	12	>50	12	>50	0	>50	1	2.048	99
3817.v2.c59	CD	>50	0	>50	0	>50	0	>50	0	>50	21
6480.v4.c25	CD	>50	15	1.264	95	5.291	87	1.05	96	0.056	100
6952.v1.c20	CD	>50	4	>50	6	>50	43	>50	3	0.051	100
6811.v7.c18	CD	>50	14	7.387	79	>50	32	22.309	65	0.117	100
89-F1_2_25	CD	>50	14	>50	15	>50	17	>50	7	>50	17
3301.v1.c24	AC	>50	7	>50	7	>50	21	>50	10	0.103	100
6041.v3.c23	AC	>50	2	0.148	99	7.19	84	0.349	98	0.018	100
6540.v4.c1	AC	>50	15	>50	15	>50	10	>50	9	>50	6
6545.v4.c1	AC	>50	9	>50	0	>50	1	>50	7	>50	12
0815.v3.c3	ACD	>50	0	>50	0	>50	36	>50	0	0.039	100
3103.v3.c10	ACD	>50	0	>50	0	>50	0	>50	0	2.16	100
MuLV	Neg. Control	>50	12	>50	10	>50	18	>50	0	>50	18
**GMT (ug/ml)**		**0.73**		**3.08**		**7.70**		**4.40**		**0.46**	
Total viruses		119		119		119		119		119	
IC50<50ug/ml		2		37		20		33		107	
**Neutralization breadth**		**1.7%**		**31.1%**		**16.8%**		**27.7%**		**89.9%**	

B

8g	
**IC50 (µg/ml)**	**MPI(%)**
0.085	100
0.148	99
0.208	99
0.209	99
0.257	100
0.335	100
0.361	99
0.438	100
0.803	97
1.03	100
1.264	95
1.55	95
1.737	85
2.015	99
2.134	95
2.429	86
3.134	97
3.279	91
3.337	86
3.379	98
4.732	83
5.512	91
7.387	79
7.426	84
8.432	81
9.327	84
10.848	81
11.487	82
11.798	73
17.561	74
18.936	69
22.793	70
25.674	59
26.957	60
33.588	58
39.835	58
40.05	58

26g	
**IC50 (µg/ml)**	**MPI(%)**
0.543	100
0.627	98
0.878	100
1.383	94
1.6494	96
2.879	95
5.029	76
5.291	87
7	90
7.19	84
8.692	86
9.821	80
19.789	62
28.147	65
28.472	59
33.548	60
40.278	58
47.389	53
48.345	51
48.734	53

41g	
**IC50 (µg/ml)**	**MPI(%)**
0.113	100
0.321	100
0.349	98
0.383	100
0.421	97
0.58	93
1	99
1.05	96
1.265	99
1.437	99
1.593	96
2.469	95
2.533	94
3.234	93
3.319	95
5.142	79
5.222	83
7.744	79
8.075	79
9.156	83
9.639	82
9.77	86
15.104	69
16.091	72
17.376	68
22.309	65
23.46	62
25.294	64
27.118	59
36.631	59
41.062	56
44.615	53
49.407	50

IC50 (µg/ml)
<0.1
0.1-1
1-10
10-50
>50

MPI(%)
100
90
80
70
60
50
40
30
20
10
0

C

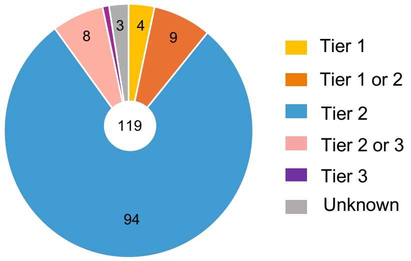

Supplement: S9 Fig — (A) The table shows the complete neutralization data of the top three IA-VRC01.v2 antibodies (8g, 26g, 41g), the original IA-VRC01 and mature VRC01. Fig 7 is based on this data. The color codes for IC50 and MPI are shown to the right. (B) The table highlights the correlation between IC50 and MPI for antibodies 8g, 26g and 41g. The data are derived from (A); the table lists only the viral isolates with IC50 < 50μg/ml. (C) Tier distribution of the 119 pseudoviruses in the neutralization panel. (PDF) [file ppat.1014373.s009.pdf]
