# Supplementary material for: Maturation of HIV-1 neutralizing antibodies in a germinal center conditional expression mouse model
Source: PLoS Pathog. 2026 Jun 22;22(6):e1014373. doi: 10.1371/journal.ppat.1014373 (PMC13313368; doi:10.1371/journal.ppat.1014373)
Supplement: S1 File — (PDF) [file ppat.1014373.s015.pdf]

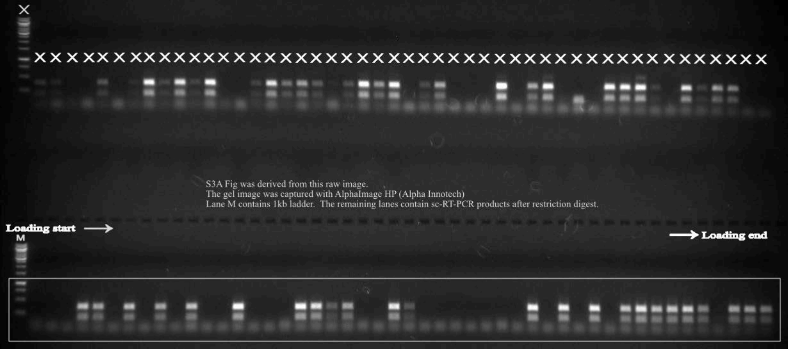
M
S3A Fig was derived from this raw image.
The gel image was captured with AlphaImage HP (Alpha Innotech)
Lane M contains 1kb ladder. The remaining lanes contain sc-RT-PCR products after restriction digest.
Loading start
Loading end
M

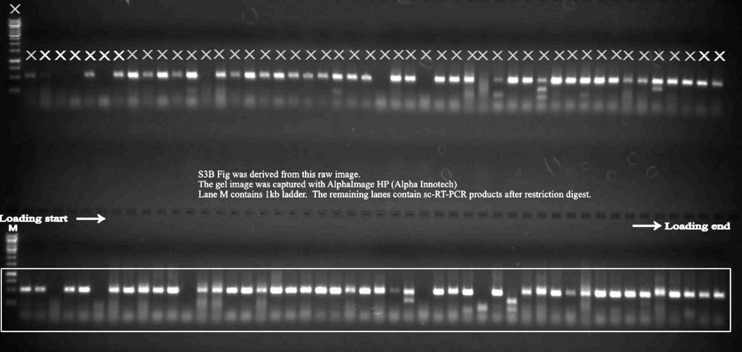
S3B Fig was derived from this raw image.
The gel image was captured with AlphaImage HP (Alpha Innotech)
Lane M contains 1kb ladder. The remaining lanes contain sc-RT-PCR products after restriction digest.
Loading start
M
Loading end

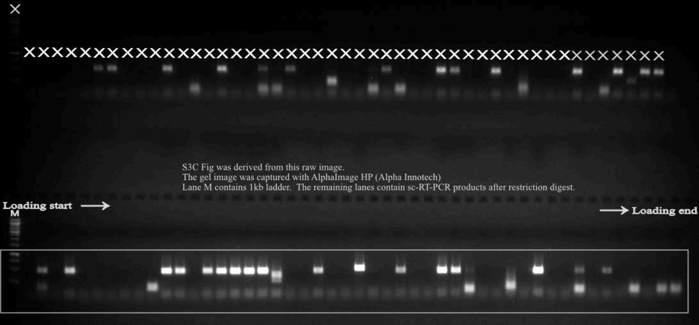
S3C Fig was derived from this raw image.
The gel image was captured with AlphaImage HP (Alpha Innotech)
Lane M contains 1kb ladder. The remaining lanes contain sc-RT-PCR products after restriction digest.
Loading start
M
Loading end

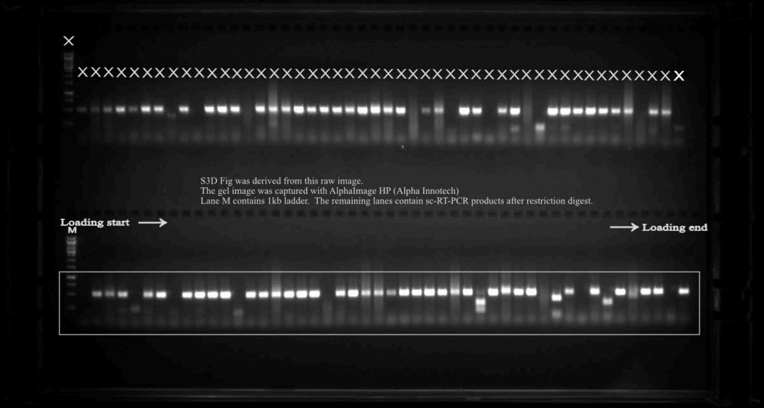

S3D Fig was derived from this raw image.
The gel image was captured with AlphaImage HP (Alpha Innotech)
Lane M contains 1kb ladder. The remaining lanes contain sc-RT-PCR products after restriction digest.
